# Peripheral T cell lymphoma after chronic lymphocytic inflammation with pontine perivascular enhancement responsive to steroids (CLIPPERS): a case report

**DOI:** 10.1186/s12883-019-1507-z

**Published:** 2019-11-04

**Authors:** Xiao-hang Liu, Fan Jin, Meng Zhang, Mei-xi Liu, Tao Wang, Bo-ju Pan, Lu Zhang

**Affiliations:** 1 Dept. of Cardiology, Peking Union Medical College Hospital, Chinese Academy of Medical Sciences & Peking Union Medical College, Beijing, 100730 China; 2 Dept. of Infectious Diseases, Peking Union Medical College Hospital, Chinese Academy of Medical Sciences & Peking Union Medical College, Beijing, 100730 China; 3 Dept. of Hematology, Peking Union Medical College Hospital, Chinese Academy of Medical Sciences & Peking Union Medical College, Beijing, 100730 China; 4 Dept. of Pathology, Peking Union Medical College Hospital, Chinese Academy of Medical Sciences & Peking Union Medical College, Beijing, 100730 China

**Keywords:** CLIPPERS, PTCL-NOS, Biopsy, Steroids

## Abstract

**Background:**

Chronic lymphocytic inflammation with pontine perivascular enhancement responsive to steroids (CLIPPERS) is an inflammatory disorder in the central nervous system (CNS) with distinct clinical, radiological, and pathological characteristics. The pathophysiology of CLIPPERS still remains unclear. Because a few cases about lymphoma mimicking the manifestations of CLIPPERS were reported and the prognosis of lymphoma is much worse, early identification of lymphoma is very important.

**Case presentation:**

A 31-year-old woman was admitted with 3 months’ history of diplopia, dizziness, gait ataxia, and right facial numbness. The diagnosis of CLIPPERS was established based on the finding of punctate enhancing lesions in the cerebellum, thalamus, pons, medulla, and midbrain region in magnetic resonance imaging (MRI), together with the favorable clinical and radiological responses to corticosteroids. However, she was diagnosed as peripheral T cell lymphoma, not otherwise specified (PTCL-NOS) by the pulmonary nodular and the skin biopsy almost 10 years later, and she got complete remission within 1 year after chemotherapy.

**Conclusion:**

We report the first case of CLIPPERS developing PTCL-NOS. This case proposes that when brain biopsy was difficult to achieve, biopsies in extra-cerebral lesions under the assisting examination of positron emission tomography-computed tomography (PET-CT) can be helpful in further identification.

## Background

Chronic lymphocytic inflammation with pontine perivascular enhancement responsive to steroids (CLIPPERS) is a treatable central nervous system inflammatory syndrome with distinct clinical, radiological, and pathological characteristics, and marked corticosteroid responsiveness [1, 2]. Recently, it has been gradually found that outcome of CLIPPERS is not necessarily benign, because some of the cases turned out to be lymphoma [3–6]. However, whether CLIPPER is an independent, new disease or a pre-lymphoma state remains unclear [7]. Now, we describe one more patient clinically diagnosed as CLIPPERS developed peripheral T cell lymphoma, not otherwise specified (PTCL-NOS), which was confirmed by extra-brain biopsy.

## Case presentation

In April 2009, a 31-year-old woman presented with a 3-month-history of progressive diplopia, dizziness, gait ataxia, and right facial numbness. Clinical examination indicated third and seventh nerve palsy, nystagmus, and bilateral vision loss. Cranial magnetic resonance imaging (MRI) revealed T2 hyperintense punctate lesions in the pons, brachium pontis, and cerebellum, with patchy and nodular enhancement in T1-weighted images. Spinal cord MRI was normal.

Various differentials for the MRI characteristics were pondered including emyelinating diseases, lymphoma, infections, vasculitis, sarcoidosis, and CLIPPERS. Cerebrospinal fluid (CSF) revealed normal protein and white blood cells count. No oligoclonal bands and malignant cells were observed. CSF etiological examination including bacterial, mycobacterium, and fungal were negative. Immunological examination including antinuclear antibodies, anti-neutrophil cytoplasmic antibodies and serum angiotensin converting enzyme were negative. Computed tomography (CT) of the pulmonary showed bilateral multiple nodules, the size of nodules was stable during regular follow-up for 4 years.

She was treated for 7 consecutive days with 20 mg intravenous dexamethasone and a tapering course of oral prednisone. The corticosteroid therapy resulted in marked neurological improvement within 7 days and the patient’s clinical findings returned to normal limits within 1 month. MRI of the brain in the following month showed dramatic improvement in radiological finding. Oral corticosteroid treatment discontinued after 6 months.

However, in October 2013, the patient again developed subacute gait ataxia, diplopia, tinnitus and right extremities weakness. Her clinical examination is now showing impaired coordination and pyramidal signs (Chaddock sign was positive). The MRI scans revealed an increased number of gadolinium-enhanced hyperintense lesions in the cerebellum, pons, medulla, and midbrain region. CSF analysis was normal. She was readmitted and treated for 4 consecutive days with intravenous methylprednisolone (500 mg once a day) followed by oral prednisone 60 mg (1 mg/kg/day) every day. The clinical findings of extremities weakness, diploma and tinnitus were improved, but there was no significant change in gait ataxia.

**Fig. 1:**
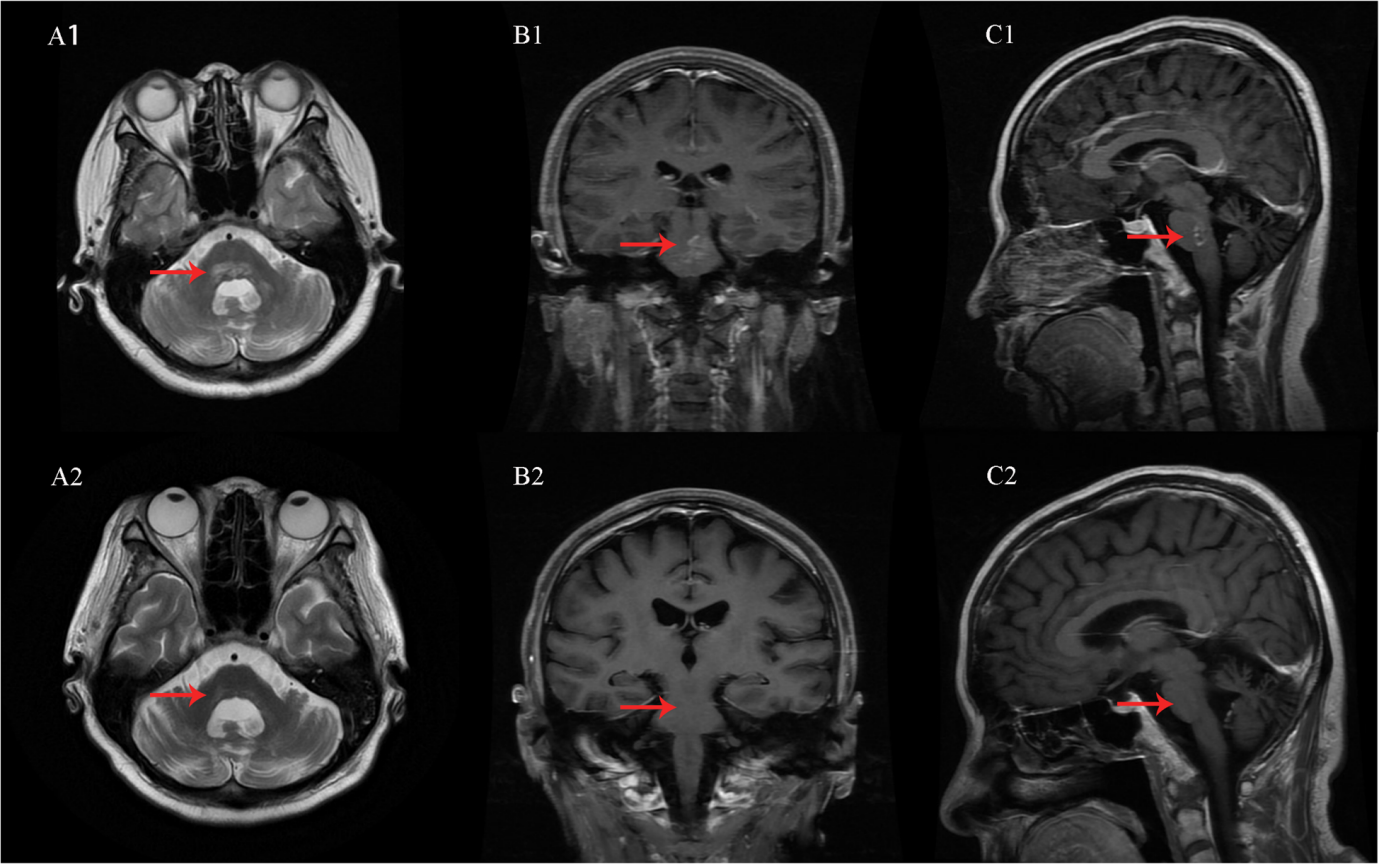
Neuroradiological images on admission and 1 month after steroid therapy. Representative axial T2-weighted imaging (A1, A2), coronal (B1, B2) and sagittal (C1, C2) T1-weighted imaging with gadolinium enhancement on brain MRI demonstrate changes of MRI features after steroid therapy. The images performed on admission show multiple punctuate hyperintense lesions within the pons, brachium pontis, and cerebellum, with a perivascular enhancement pattern (red arrow, top row). A decrease in the number and extent of pathology is observed on a brain MRI after steroid treatment (red arrow, bottom row)

Unfortunately, only 2 months after treatment with a tapering course of oral prednisone (prednisone was reduced to 15 mg at that time), our patient presented with symptoms similar to those in the last admission again, in addition to left extremities tinnitus. By then, brain biopsy was considered to be risky given the deep location of the lesion, and our patient was reluctant to take the risk of doing this examination. Therefore, we didn’t do brain biopsy for further confirmation. Although a definitive diagnosis could not be established, because the radilogical findings and good therapeutic response to glucocorticoid were suggestive, a working diagnosis of CLIPPERS was made. She was treated for 5 consecutive days with intravenous methylprednisolone (1000 mg once a day) followed by oral prednisone (60 mg every day). Treatment resulted in significant clinical improvement of symptoms within a week, and MRI scans showed a dramatic decrease in the number and extent of gadolinium-enhanced lesions **(**Fig. 1**)**.

**Fig. 2:**
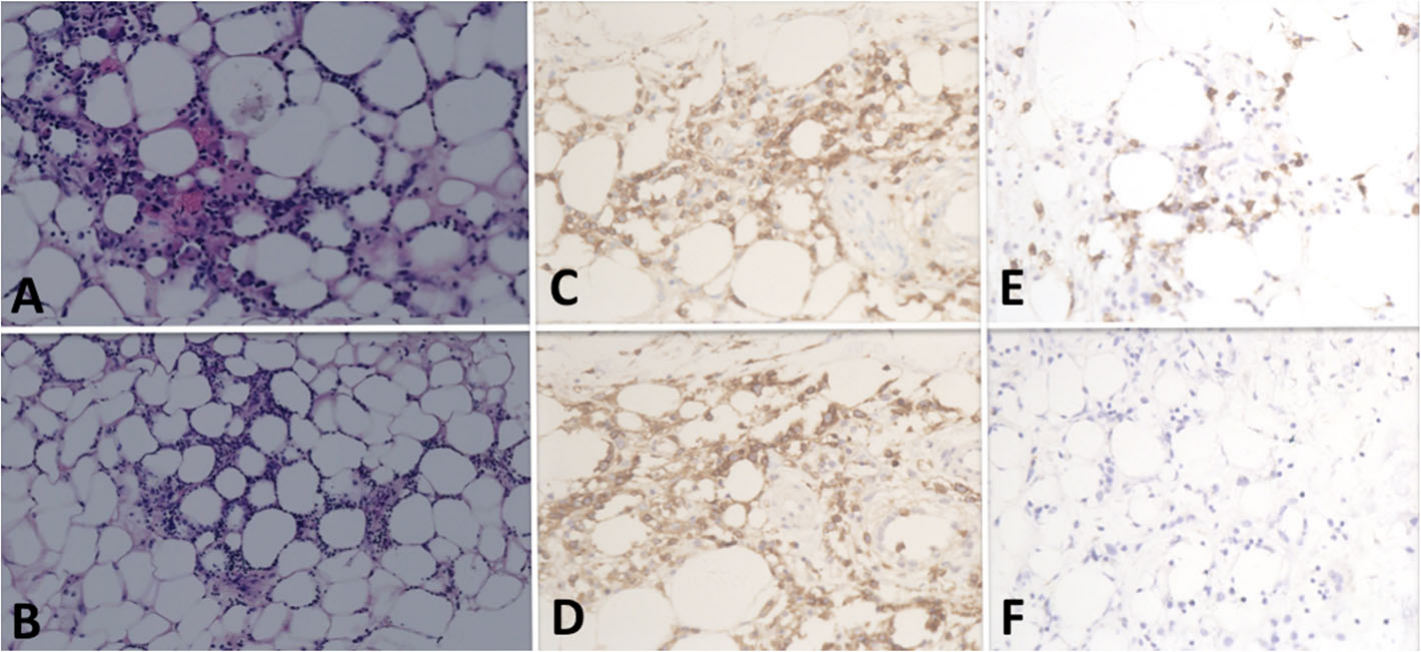
Skin biopsy from abdomen showing subcutaneous infiltrate pleomorphic, small-sized atypical lymphocytes (H&E, **a**, original magnification × 200; **b**, original magnification × 150;). Lymphocytes predominantly positive for: CD3 (**c**), CD4 (**d**), CD8 (**e**), CD56 (**f**) (original magnification: × 200)

In February 2018, she developed fever and pancytopenia. Positron emission tomography-computed tomography (PET-CT) revealed abnormal accumulation of fluourodeoxyglucose in the pulmonary nodular lesions and the whole body skin. Pathological of lung biopsy revealed accumulation of pleomorphic, small-sized atypical lymphocytes. Immunohistochemistry showed proliferative cells were positive for CD3 and CD5. Rearranged T-cell receptor γ-chain and β-chain genes analyzed by polymerase chain reaction (PCR) were negative. Finally, peripheral T cell lymphoma, not otherwise specified (PTCL-NOS) was diagnosed. The abdomen skin biopsy further demonstrated PTCL-NOS predominantly comprised of CD3, CD4, CD8 and CD56-positive T-cells **(**Fig. 2**)**. Therefore, it was classified as Stage IVB lymphoma in the Ann Arbor classification. She got complete remission after 4 cycles of the ECHOP regimen (etoposide, cyclophosphamide, doxorubicin, vincristine, and prednisone) in September 2018.

## Discussion and conclusions

CLIPPERS was first reported by Pittock et al. in 2010 [8]. It is an inflammatory syndrome of the central nervous system (CNS), prominently involving the brainstem and pons. The clinical manifestations of CLIPPERS are characterized by gait ataxia, dysarthria, diplopia, and altered facial sensation. The magnetic resonance imaging (MRI) shows a punctate and nodular pattern of gadolinium enhancement “peppering” in the pons, brainstem, white matter, and other adjacent structures. Perivascular lymphohistiocytic and predominant T cells infiltration in brain biopsy specimen is another striking characteristic. As the name implies, CLIPPERS patients present a favorable and dependent response to steroids therapy [1–3]. Our patient was admitted with a long history of CNS syndrome, especially gait ataxia. The MRI features, along with the favorable clinical and radiological responses to corticosteroids led to the diagnosis as “probable CLIPPERS” when lacking neuropathological examination according to the diagnostic criteria updated in 2017 by W. Oliver Tobin et al. [2].

It is important to note that, infectious etiologies, paraneoplastic, neoplastic, and dominant inflammatory processes can mimick CLIPPERS. Moreover, some of them are malignant and rapidly deteriorating [1]. For example, Berkman and colleagues reported a patient with typical clinical and radiographic characteristics of CLIPPERS who was finally diagnosed as Erdheim–Chester disease (ECD) by brain biopsy [9]. Since Limousin and colleagues reported a case with clinical manifestation of CLIPPERS but final diagnosis of type B primary CNS lymphoma in 2011, [4] several cases of patients with CLIPPERS associated with lymphoma followed. However, only two patients of CLIPPERS associated with T cell lymphoma have been reported [5, 6]. One case of cutaneous T-cell lymphoma (Mycosis fungoides) [5] and one case of PTCL-NOS, [6] were both presenting eventually with CLIPPERS features. We reported the first case presenting initially with CLIPPERS of clinical and radiological features progressed eventually to PTCL-NOS. Because of the high concern for more malignant processes, the exclusion strategy of the CLIPPERS diagnosis must be paid great attention to in order to avoid an incorrect diagnosis [1, 2].

It is true that brainstem biopsy plays an important part for accurate histologic diagnosis in CLIPPERS, but sometimes the potential complications including hemorrhage, intracranial hypertension and edema limit its utility [10]. In our case, the patient was finally diagnosed as PTCL-NOS by the pulmonary nodular and the skin biopsies. She was timely treated with chemotherapy and obtained complete remission. This suggests that, when a clinical diagnosis of CLIPPERS is highly suspicious and a brain biopsy could not be done for some reason, biopsies in extra-cerebral lesions can be alternative options. For example, hypermetablic lesions in PET-CT may help locate the biopsy parts. The patient in our study found hypermetablic lesions in bilateral lungs and skin after PET-CT and the following biopsy confirmed lymphoma.

In summary, lymphoma can mimick CLIPPERS in clinical manifestations. The present case proposes that biopsies in extra-cerebral lesions under the assisting examination of PET-CT can be helpful in further identification. Further studies are required to determine the relationship between lymphoma and CLIPPERS and the potential value of systemic imaging in discriminating lymphoma from CLIPPERS during the early stages of the disease and during the follow-up period.

## Data Availability

All data related to this case report are contained within the manuscript.

## Abbreviations

CLIPPERS: Chronic lymphocytic inflammation with pontine perivascular enhancement responsive to steroids
CNS: Central nervous system
CT: Computed tomography
ECHOP: Etoposide, cyclophosphamide, doxorubicin, vincristine, prednisone
MRI: Magnetic resonance imaging
PCR: Polymerase chain reaction
PET-CT: Positron emission tomography-computed tomography
PTCL-NOS: Peripheral T cell lymphoma, not otherwise specified
